# Smartphone-Based Self-Monitoring, Treatment, and Automatically Generated Data in Children, Adolescents, and Young Adults With Psychiatric Disorders: Systematic Review

**DOI:** 10.2196/17453

**Published:** 2020-10-29

**Authors:** Sigurd Melbye, Lars Vedel Kessing, Jakob Eyvind Bardram, Maria Faurholt-Jepsen

**Affiliations:** 1 The Copenhagen Affective Disorder Research Center (CADIC) Psychiatric Centre Copenhagen, Rigshospitalet København Ø Denmark; 2 Department of Applied Mathematics and Computer Science The Technical University of Denmark Lyngby Denmark

**Keywords:** mHealth, child and adolescent psychiatry, eHealth, systematic review, psychiatry, mobile phone

## Abstract

**Background:**

Psychiatric disorders often have an onset at an early age, and early identification and intervention help improve prognosis. A fine-grained, unobtrusive, and effective way to monitor symptoms and level of function could help distinguish severe psychiatric health problems from normal behavior and potentially lead to a more efficient use of clinical resources in the current health care system. The use of smartphones to monitor and treat children, adolescents, and young adults with psychiatric disorders has been widely investigated. However, no systematic review concerning smartphone-based monitoring and treatment in this population has been published.

**Objective:**

This systematic review aims at describing the following 4 features of the eligible studies: (1) monitoring features such as self-assessment and automatically generated data, (2) treatment delivered by the app, (3) adherence to self-monitoring, and (4) results of the individual studies.

**Methods:**

We conducted a systematic literature search of the PubMed, Embase, and PsycInfo databases. We searched for studies that (1) included a smartphone app to collect self-monitoring data, a smartphone app to collect automatically generated smartphone-based data, or a smartphone-based system for treatment; (2) had participants who were diagnosed with psychiatric disorders or received treatment for a psychiatric disorder, which was verified by an external clinician; (3) had participants who were younger than 25 years; and (4) were published in a peer-reviewed journal. This systematic review was reported in accordance with the Preferred Reporting Items for Systematic Reviews and Meta-Analyses guidelines. The risk of bias in each individual study was systematically assessed.

**Results:**

A total of 2546 unique studies were identified through literature search; 15 of these fulfilled the criteria for inclusion. These studies covered 8 different diagnostic groups: psychosis, eating disorders, depression, autism, self-harm, anxiety, substance abuse, and suicidal behavior. Smartphone-based self-monitoring was used in all but 1 study, and 11 of them reported on the participants’ adherence to self-monitoring. Most studies were feasibility/pilot studies, and all studies on feasibility reported positive attitudes toward the use of smartphones for self-monitoring. In 2 studies, automatically generated data were collected. Three studies were randomized controlled trials investigating the effectiveness of smartphone-based monitoring and treatment, with 2 of these showing a positive treatment effect. In 2 randomized controlled trials, the researchers were blinded for randomization, but the participants were not blinded in any of the studies. All studies were determined to be at high risk of bias in several areas.

**Conclusions:**

Smartphones hold great potential as a modern, widely available technology platform to help diagnose, monitor, and treat psychiatric disorders in children and adolescents. However, a higher level of homogeneity and rigor among studies regarding their methodology and reporting of adherence would facilitate future reviews and meta-analyses.

## Introduction

### Background

Psychiatric disorders often have an onset of symptoms at an early age, and 3 out of 4 patients with mental disorders have an onset of symptoms before the age of 24 years [1]. According to the World Health Organization, mental health problems account for 16% of the global burden of disease in people aged 10-19 years [2]. For these patients, the early identification of symptoms and interventions may potentially lead to significant improvement in their quality of life, level of function, sense of empowerment, and prognosis [3].

Currently, markers such as blood tests, radiologic findings, or electrophysiological measurements are insufficient for supporting the diagnostic assessment of psychiatric disorders and the severity of the symptoms. Diagnoses are largely based on clinical evaluations and observations; therefore, the affected children may depend on parents/relatives/support systems and their ability to accurately report symptoms. A fine-grained, unobtrusive, and effective way to monitor symptoms and function could help distinguish severe psychiatric health problems from normal behavior and potentially lead to a more efficient use of clinical resources in today’s health care system, which in turn can lead to a more equitable distribution of resources.

Ecological momentary assessment, which involves repeatedly sampling a subject’s current behaviors and experiences in real-time in his/her natural environments, reduces potential recall bias and is able to give a valid momentary overview of the fluctuation of symptoms and the level of function [4]. Smartphones represent a promising platform for ecological momentary assessments, as they are readily available to many people worldwide [5]. For adolescents and young adults, interaction with a smartphone is a natural part of everyday life, and a report from the Pew Research Centre shows that 95% of the teens in the United States own a smartphone [6]. Automatically generated data collected from smartphones and wearable sensors can be combined with detailed information on the physical health, mental health, and behaviors of children and young adults to potentially aid in diagnosing, monitoring, and treating psychiatric disorders. Thus, smartphones represent a promising tool to unobtrusively obtain access to momentarily continuous data.

Smartphone apps are also widely used as a platform to deliver treatments to users with mental health disorders and may offer an alternative to patients who have difficulties participating in traditional face-to-face therapy. Furthermore, smartphone apps are able to deliver treatment between outpatient visits, thereby enabling early intervention when prodromal symptoms or signs of deterioration begin to present. However, very few apps deliver content that is in line with evidence-based theories; in a systematic review from 2019 on apps that deliver cognitive behavioral therapy and behavioral activation, only 12 out of 107 apps were consistent with evidence-based principles [7]. Even though many apps report high feasibility and user satisfaction, very few studies have investigated the clinical effects of this technology [8]. Nevertheless, over the past few years, an increasing number of studies have investigated the use of smartphone apps to monitor and treat children, adolescents, and young adults with psychiatric symptoms. However, to date, no systematic review exists on the use of smartphones for monitoring and treatment of symptoms in children, adolescents, and young adults clinically diagnosed with psychiatric disorders. In this systematic review, we wanted to restrict our inclusion to studies involving individuals diagnosed with a psychiatric disorder in order to allow the findings to be generalizable to clinical populations.

### Aim of This Study

The overall aim of this systematic review was to present the overview and status of studies investigating the use of smartphones for self-monitoring, treatment, or automatically generated data (eg, smartphone usage or location tracking) in children, adolescents, and young adults with psychiatric disorders. In particular, we aimed to conduct a systematic review that identifies and evaluates all of the studies on children, adolescents, or young adults who have been clinically diagnosed with a psychiatric disorder that include the smartphone-based self-monitoring of symptoms and level of function or smartphone-based treatment intervention. Additionally, we aimed to describe the following features of the eligible studies: (1) monitoring features such as self-assessment and automatically generated data, (2) content of the treatment delivered by the app, (3) adherence of the participants to self-monitoring, and (4) results of individual studies.

## Methods

### Design

This systematic review was reported according to the Preferred Reporting Items for Systematic Reviews and Meta-Analysis (PRISMA, Multimedia Appendix 1) [9]. The eligibility criteria and search methodology were established and documented in advance by 3 of the authors (SM, LVK, MFJ). During the review process, we decided to also include studies that only used smartphone technology to deliver treatment in addition to studies that used smartphones for monitoring, as was the original criterium.

### Study Selection

The definitions of children, adolescents, and young adults may differ depending on the culture or tradition. The World Health Organization defines “young people” to be individuals between the ages of 10 and 24 years [10]. A “child” is defined as a person younger than 18 years, and the term “adolescents” is used to describe individuals between the ages of 10 and 19 years [10]. In this review, we chose to define children, adolescents, and young adults as individuals younger than 25 years. For the papers included in this review, the following inclusion criteria were applied: (1) the study utilized a smartphone app to collect self-monitoring data or automatically generated data such as step counts, phone usage, and location data, or the study used a smartphone-based system for treatment; (2) the participants were referred by a clinician who already provided a psychiatric disorder diagnosis, or they received treatment for the disorder, or had severe symptoms requiring treatment, for example, suicidal behavior, self-harm behavior; (3) the participants were 25 years or younger or the vast majority of the participants in the study were younger than 25 years, which was reflected by a low mean age; and (4) the study was published in a peer-reviewed journal. For studies that were described by several papers, the most recent paper was chosen for inclusion in this review. During the review process, the inclusion criteria concerning the diagnostic foundation of the participants were clarified. Precisely, we added “participants were referred by a clinician who already provided a psychiatric disorder diagnosis, or they received treatment for the disorder” to criteria (2). This was done because we found several studies including participants who only self-reported that they received treatment owing to severe symptoms. Thus, we found the initial criteria to not be sufficiently precise regarding the clinical status of the participants to identify all the relevant studies for the review. The exclusion criteria were as follows: (1) the studies included people with symptoms not meeting the diagnostic criteria or who only self-reported symptoms and were not referred by a clinician; (2) the paper was an abstract, systematic review, case report, or protocol; and (3) the paper was not written in English.

### Search Strategy

Studies were selected for inclusion in this review through a systematic search of the PubMed, PsycInfo, and Embase databases on May 25, 2020, for all studies published prior to this date. The following search string was designed to target studies that included children, adolescents, or young adults with psychiatric disorders and the smartphone-based registration of symptoms: (adolescents OR young adults OR young OR teenagers OR children) AND (drug OR substance OR prescription drug OR alcohol OR narcotic OR heroin OR cocaine OR amphetamine OR cocaine OR marijuana OR opioid OR morphine OR phencyclidine) AND (abuse OR dependence OR addiction) OR (feeding disorder OR feeding disorders OR eating disorders OR eating disorder OR anorexia OR bulimia OR binge eating) OR (autism OR autistic OR asperger disease OR aspergers disease) OR asperger disorder OR aspergers disorder OR adhd OR attention deficit disorder OR attention deficit hyperactivity disorder OR (personality disorder OR personality disorders OR obsessive-compulsive personality OR compulsive personality OR obsessive personality OR psychopath OR sociopathic OR antisocial OR passive-dependent personality OR dissocial OR schizoid OR schizotypal) OR (schizophrenia OR psychoses OR psychosis OR psychotic OR paranoid OR schizoaffective OR schizophreniform OR delusional) OR (major depressive disorder OR unipolar depression OR unipolar disorder OR depressive syndrome OR endogenous depression OR neurotic depression OR melancholia OR cyclothymic OR dysthymic OR mood disorder OR mood disorders OR affective disorder OR affective disorders OR bipolar OR manic-depressive OR mania OR manic) OR (anxiety OR anxieties OR panic disorder OR agoraphobia OR obsessive disorder OR compulsive disorder OR obsessive-compulsive disorder OR phobic disorder OR phobic disorders OR ptsd OR posttraumatic stress disorder OR posttraumatic stress disorder OR posttraumatic stress disorder) AND (smartphone OR cellphone).

In order to include studies published within the last 6 months, which were not yet indexed by keywords, a literature search was conducted using the Text Word field tag in PubMed. In Embase, the field tag Keywords were used, and in PsycInfo, the field tag All Text was used. There were no limits applied to the search. We did not conduct a grey literature search.

### Study Selection and Data Extraction

The identified studies were imported into EndNote for further processing. After importing the studies, duplicates were removed—first automatically and then manually. Studies were then screened for eligibility by SM. For each study, the following data were extracted: (1) general description of the study: author(s), year of publication, country, sample size, study design, age of population, sex of participants, clinical profile of case group, and the follow-up period of the study; (2) description of the app: name of the app, operating system in the app, items in self-monitoring, items collected in automatically generated data, and whether the app delivered treatment; (3) treatment delivered by the app: description of the intervention; and (4) description of the control group, study procedure, and findings: description of the control group, baseline assessment, number of follow-ups, adherence to self-monitoring, and the findings.

The data extraction was performed by SM and validated by MFJ. Any disagreements or uncertainties regarding eligibility or data to be extracted were resolved by discussion between 3 researchers (SM, MFJ, and LVK). The randomized controlled studies in this review were assessed for risk of bias by SM by using the Cochrane Risk of Bias tool [11]. For the remaining studies, the quality of the evidence was assessed using the GRADE (Grading of Recommendations, Assessment, Development and Evaluations) guidelines [12].

## Results

### Study Selection

**Figure 1:**
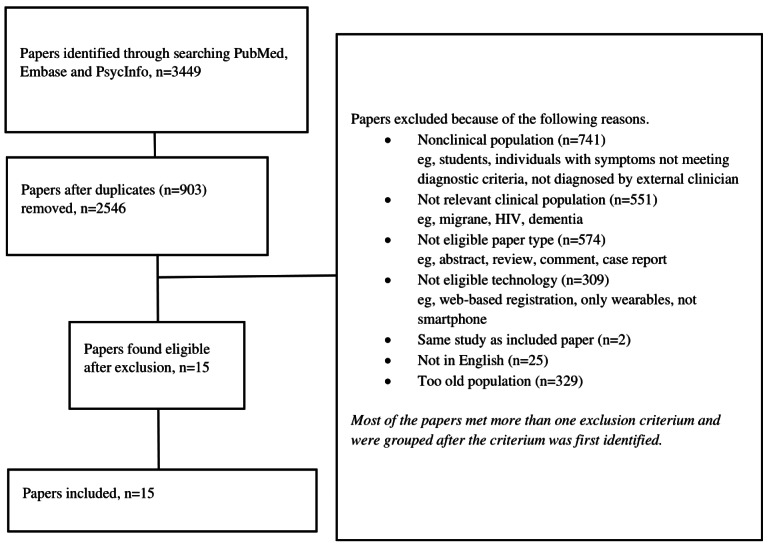
PRISMA (Preferred Reporting Items for Systematic Reviews and Meta-Analysis) flow diagram displaying information on study flow from initial search to final inclusion.

The search resulted in the identification of 3449 studies. After duplicates were removed, 2562 unique studies were screened either by title, abstract, or full text. The majority of the studies fell under the exclusion criteria such as nonclinical population (eg, students, individuals with subsyndromal symptoms, and individuals recruited via social media/flyers), population out of the defined age group, technology not delivered by a smartphone app (eg, web-based or use of only wearables), and publication type other than full-text paper published in a peer-reviewed journal. Finally, a total of 15 papers describing 15 unique studies were included for the review. The study selection process is presented as a PRISMA flow diagram in Figure 1.

### Diagnoses and Study Origin

Of the 15 papers describing 15 unique studies (Table 1), 4 were concerning participants with psychosis [13-16], 3 were concerning participants with eating disorders [17-19], 2 were concerning participants with depression [20,21], 2 were concerning participants with autism [22,23], 1 was concerning participants with substance abuse [24], 1 was concerning participants with suicidal behavior [25], 1 was concerning participants with self-harming behavior [26], and 1 was concerning participants with anxiety [27]. In all the included studies, participants were referred by external clinicians who had established the diagnosis or the need for treatment, and in 2 studies, the diagnoses were also validated by researchers at baseline [18,27]. Three studies had some participants older than 25 years but the clear majority of the participants were children, adolescents, or young adults, as demonstrated by the low mean age [14,15,19]. Four studies included only females [17-20], 2 did not provide information about gender [22,23], while the remaining studies included both genders. Of the 15 studies, 11 were published in 2017 or later [13-16,19,20,22,25-27]. A total of 7 studies originated from the United States [14,15,20-22,24,25], 5 from Europe [13,17-19,26], 2 from Australia [16,27], and 1 from Jordan [23].

**Table 1:** Description of the study design and populations of studies on self-monitoring and automatically generated data collected via smartphones in children, adolescents, and young adults with psychiatric disorders (N=15).

Author, year of publication	Country	Sample size (n)	Study design	Age of population (years), range, mean (SD)/proportion^a^	Sex (women), (%)	Clinical profile of the case group	Length of project
Bucci et al, 2018 [13]	United Kingdom	24 in intervention arm, 12 in control arm	Randomized controlled trial	≥16 years, age at first symptoms: Actissist group, 20.21 (7.37) years, ClinTouch group, 18.33 (7.00) years	Actissist group: 37.5%, ClinTouch group: 75.0%, Total: 50%	Early psychosis	12 weeks
Cao et al, 2020 [21]	United States of America	13	Feasibility study	12-17 years, 14.9 (1.59) years	85%	Depression	8 weeks
Dennis et al, 2015 [24]	United States of America	29	Feasibility study	14-15 years, 28% 16-17 years, 45% 18 years, 28%	31%	Substance abuse	6 weeks
Jones et al, 2018 [22]	United States of America	20	Feasibility study	5-13 years	Not described	Autism	8 weeks
Kennard et al, 2018 [25]	United States of America	34 in intervention arm, 32 in control arm	Randomized controlled trial	12-18 years, intervention group: 14.9 (1.6) years; control group: 15.3 (1.4) years	Intervention group: 90.6%; control group: 88.2%	Suicidal ideation or recent suicide attempt	2-3 weeks
Kolar et al, 2016 [17]	Germany	20 cases, 20 healthy controls	Observational study	12-19 years, cases: 16.0 (1.55) years; controls: 15.9 (1.95) years	100%	Anorexia nervosa	2 days
Kumar et al, 2018 [14]	United States of America	61	Feasibility study	12-30 years, mean 17.4 years	48.50%	Early psychosis	Up to 5 months
Lim et al, 2020 [16]	Australia	12	Feasibility study	16-25 years, 20.50 (1.33) years	25%	Early psychosis	6 weeks
Lim et al, 2019 [27]	Australia	9 cases, 11 healthy controls	Feasibility study	18-23 years, case group: 21.00 (1.41) years; control group: 20.36 (2.16) years; total: 20.65 years	44.99%	Social anxiety disorder	6 weeks
Neumayr et al, 2019 [19]	Germany	20 in intervention arm, 20 in control arm	Randomized controlled trial	15-36 years, intervention group, 20.75 (6.4) years; control group, 18.00 (3.73) years	100%	Anorexia nervosa	8 weeks
Niendam et al, 2018 [15]	United States of America	76	Feasibility study	13-30 years, 18.8 (3.7) years	44%	Recent onset psychosis and clinical high risk	3-14 months
Seidel et al, 2016 [18]	Germany	37 cases, 33 healthy controls	Retrospective cohort	Cases: 12-20 years, 16.40 (2.33) years; control: 14-25 years, 16.51 (3.79) years	100%	Anorexia nervosa	2 weeks
Shrier and Spalding, 2017 [20]	United States of America	16	Feasibility study	15-23 years, mean 19.6 years	100%	Depression and sexual risk behavior	4 weeks
Stallard et al, 2018 [26]	United Kingdom	44	Feasibility study	12-17 years, 16.0 (1.4) years	91%	Self-harming or history of self-harm	12 weeks
Sweidan et al, 2019 [23]	Jordan	100	Feasibility study	5-13 years	Not described	Autism	1 month

^a^In some studies, only the mean age/age range/mean (SD) age/all of these were provided.

### Study Characteristics

Of the 15 included studies, 3 were RCTs [13,19,25] investigating the effect of smartphone-based treatment interventions, 1 was a retrospective cohort study [18], 1 was an observational study [17], and the remaining were feasibility/pilot studies. The sample sizes of the included studies varied from 12 [16] to 100 [23] participants, with a mean (SD) sample size of 42.9 (26.5) participants.

### Technical Description of the Smartphone Technology

One of the studies used an app that only administered treatment and did not use a monitoring system [23], 8 studies used a monitoring system and administered treatment [13,16,19,20,24-27], and the remaining 6 studies included monitoring only (Table 2). In the 15 studies, there were 14 different smartphone apps, as 1 of them was used in 2 different studies [16,27]. Six of the smartphone apps were available for Android phones only [13,17,18,21,23,24], and 6 were available for both Android phones and iPhones [14-16,19,20,22,25-27]. In 1 study, only the caregiver of the diagnosed child used the app [22]. Three studies described a design wherein clinicians used the registered data in clinical sessions [14,15,19].

**Table 2:** Description of the app used in studies on self-monitoring, treatment, and automatically generated data collected via smartphones in children, adolescents, and young adults with psychiatric disorders (N=15).

Author, year of publication	Name of app	System	Items in self-monitored data	Items in automatically generated data	Active treatment delivered by app
Bucci et al, 2018 [13]	Actissist and ClinTouch	Android	Actissist: self-assessment focused on cognitive appraisals, belief conviction, emotions, and associated behaviors. ClinTouch: rating of 12 symptoms validated against PANSS^a^	N/A^b^	Yes
Cao et al, 2020 [21]	SOLVD	Android	Daily: mood and anxiety	Accelerometer, GPS, steps, call log, text messages, screen on/off, and ambient light intensity	No
Dennis et al, 2015 [24]	ACHESS	Android	EMA^c^ 6 times/day focused on current feelings, activities, location, and company, internal and external factors that made them want to use drugs/alcohol, and their ability to resist	N/A	Yes
Jones et al, 2018 [22]	Janssen Autism Knowledge Engine	Android and iOS^d^	By caregiver: questions about the child being tense/worried, irritable, and disruptive. Once a day in weeks 1, 4, and 8, and 3 times a week in the remaining period	N/A	No
Kennard et al, 2018 [25]	BRITE	Android and iOS	Level of emotional distress	N/A	Yes
Kolar et al, 2016 [17]	Epicollect	Android	Assessment of aversive tension and possible moderator events every hour for 2 days, except predefined sleeping hours	N/A	No
Kumar et al, 2018 [14]	RealLife Exp	Android and iOS	Daily questions on mood, medication use, socialization, conflict, and medication. Weekly survey on how often in the past week they felt a range of symptoms	N/A	No
Lim et al, 2020 [16]	+Connect^e^	Android and iOS	Mood evaluation tracker	N/A	Yes
Lim et al, 2019 [27]	+Connect	Android and iOS	Mood evaluation tracker	N/A	Yes
Neumayr et al, 2019 [19]	Recovery Record	Android and iOS	Self-monitoring of meals, feelings, behavior, and thoughts.	N/A	Yes
Niendam et al, 2018 [15]	Ginger.io	Android and iOS	Daily surveys assessing mood, medication adherence, and social interactions; weekly surveys assessing symptoms, sleep, and medication adherence	Number of calls in/out, messages in/out, movement patterns based on GPS data.	No
Seidel et al, 2016 [18]	MovisensXS	Android	Rumination about food and weight; an adapted version of the MDMQ^f^ assessed 3 dimensions of affect: valence, calmness, and energetic arousal	N/A	No
Shrier and Spalding, 2017 [20]	Not described	Android and iOS	EMI^g^ regarding feeling, social situations, and sexual behavior 4 times/day. Questions about motivation to change risk behavior, stressful events, and use of healthy ways to manage feelings	N/A	Yes
Stallard et al, 2018 [26]	BlueIce	Android and iOS	Mood diary	N/A	Yes
Sweidan et al, 2019 [23]	AIA^h^	Android	N/A	N/A	Yes

^a^PANSS: Positive and Negative Syndrome Scale.

^b^N/A: not applicable.

^c^EMA: ecological momentary assessment.

^d^iOS: iPhone operating system.

^e^This same app was used in 2 studies.

^f^MDMQ: multidimensional mood questionnaire.

^g^EMI: ecological momentary intervention.

^h^AIA: Autistic Innovative Assistant.

### Smartphone Usage

The period of use for the smartphone app varied from 2 days [17] to up to 14 months [15]. For the studies where the duration was precisely defined, the mean (SD) duration was 6.1 (3.6) weeks. In 6 of the included studies, participants received financial compensation [14-16,19,24,27]; in 2 studies, patients received gift cards as compensation for participation [13,20]; 1 study reports compensating participants but does not state how [21], and in the remaining studies, information concerning economic compensation for participation was not provided.

Only 4 studies [13,16,26,27] reported that they monitored for potential adverse effects. One of the studies listed hospital admission as a potential adverse effect [16], 1 study listed increased self-harm as an adverse effect [26], 1 listed both admission and self-harm as adverse effects [27], and the last study did not specify the adverse effects that were being monitored [13]. None of these studies identified events of adverse effects during their study periods. For the remaining 11 studies, no potential adverse effects were mentioned. There were no other reported negative consequences to using the technology in any of the studies.

### Smartphone-Based Self-monitoring

All but 1 [23] of the included studies had elements of self-monitoring collected via smartphones, and self-assessment of symptoms and level of function relevant to the specific clinical population were the most frequent items included. A total of 6 studies described the self-monitoring of the participant’s mood [14-16,21,26,27], 1 study requested the participants to perform self-monitoring related to recreational drug use [24], 1 requested self-monitoring on medication adherence [15], and 1 described self-monitoring of meals [19]. In studies where the frequency of self-monitoring was specified, it varied from once a day to every waking hour [27]. One of the studies reported validating smartphone-based self-monitored data on mood and anxiety by investigating the correlation between these and the validated clinical ratings on the Hamilton Depression Rating Scale [28] and the Hamilton Anxiety Rating Scale [29]. The items used for self-monitoring in each of the studies are presented in Table 2.

### Automatically Generated Smartphone-Based Data

A total of 2 studies described the collection of automatically generated data via smartphones [15,21]. Both these studies described the collection of usage data, such as the number of phone calls and text messages in addition to GPS-based location data that provided data about the user’s movement patterns. One of the studies also collected the information on the number of steps, the amount of time the screen was turned on time, and the registered ambient light every second minute [21]; it also investigated the correlation between the automatically generated smartphone-based data and clinical findings from rating scales and found significantly positive correlations between daily steps taken, SMS frequency, and the average call duration and scores from the clinical rating scales [21]. The other study did not describe how they used the automatically generated data [15].

### Adherence to Self-monitoring

All but 4 [20,22,23,26] of the studies reported on the adherence to smartphone-based self-monitoring in some way. However, the level of adherence and acceptance was reported differently across the various studies, making it impossible to conduct meta-analyses investigating the differences in adherence measures between diagnostic categories. In 9 studies, adherence to self-monitoring was reported as a percentage—either as a percentage of the participants reaching a predefined level of satisfactory completion or as a percentage of prompts/notifications the participants responded to [13-16,18,21,24,25,27]. In all but 1 of these studies [14], the adherence to self-monitoring was above 50%. The specific rates of adherence are presented in Table 3.

**Table 3:** Description of the control groups, procedures, and findings in studies on self-monitoring, treatment, and automatically generated data collected via smartphones in children, adolescents, and young adults with psychiatric disorders (N=15).

Author, year of publication	Control group	Baseline assessment	Follow-up	Adherence	Findings
Bucci et al, 2018 [13]	24 participants received Actissist plus TAU^a^, 12 received ClinTouch plus TAU	Demographics; PANAS^b^; PSYRATS^c^; CDSS^d^; Global Assessment of Functioning Scale; Personal and Social Performance Scale; Empowerment Rating Scale; EQ-5D-5L^e^; Timeline Followback, Medication Adherence Rating Scale	Clinical assessment at 12 weeks and 22 weeks	Data points completed (>33% data entries): 75% (Actissist) and 50% (ClinTouch)	Actissist was feasible, acceptable (90% recommend Actissist), and safe (0 serious adverse events), with high levels of user satisfaction. Treatment effects were large on negative symptoms, general psychotic symptoms, and mood. The addition of Actissist conferred benefit at posttreatment assessment over routine symptom-monitoring and TAU.
Cao et al, 2020 [21]	N/A^f^	Mini International Neuropsychiatric Interview to confirm diagnosis of MDD^g^, PHQ-9^h^, HAM-D^i^, and HAM-A^j^	Biweekly clinical assessment	79.0%	Significant correlation between the self-evaluated mood averaged over a 2-week period and the biweekly psychometric scores from PHQ-9, HAM-D, and HAM-A (0.45≤|r|≤0.63; *P*=.009, *P*=.01, and *P*=.003, respectively). The daily steps taken, SMS frequency, and average call duration were also highly correlated with clinical scores (0.44≤|r|≤0.72; all *P*<.05). By combining self-evaluations and smartphone sensor data, they could predict the PHQ-9 score with an accuracy of 88%.
Dennis et al, 2015 [24]	N/A	GAIN-Q3^k^	2 visits a week to complete survey and provide urine sample	Participants completed 89% EMAs^l^; 18 participants completed over 90% of the EMAs	EMA observations were classified into 3 risk groups: “Current Use” (3%), “Unrecognized Risk” (42%), or “Recognized Risk” (55%). Unrecognized Risk (50%) and Current Use (96%) groups reported significantly higher rates of use in the next week compared with the Recognized Risk group’s use in the following week (31%). Drug use following an EMA that was accessed was lower compared to that when EMA was not accessed (32% vs 43%).
Jones et al, 2018 [22]	N/A	Caregivers completed aberrant behavior checklist, child behavior checklist, PANAS, visual analog scale	Clinic visits in weeks 1, 4, and 8.	Not described	Over 8 weeks, caregivers reported improvements in their child’s mood, irritability, and disruptive behaviors during TAU.
Kennard et al, 2018 [25]	34 patients received As Safe As Possible app+ TAU, 32 received TAU	PHQ, SIQ-JHSV^m^, CSSRS^n^, youth self-report scale, CRAFT^o^	At weeks 4, 12, and 24	70.6% used the app at least once. Participants rated their mood at a median of 19 times	There were no treatment effects on suicidal ideation. Participants reported high satisfaction with both the intervention and the app.
Kolar et al, 2016 [17]	Healthy controls	ChEDE^p^; Symptom Checklist 90	Not described	1030 completed the observations entered	Participants with anorexia nervosa showed higher mean and maximum levels of aversive tension. Reported food intake was associated with higher levels of aversive tension in the anorexia nervosa group, whereas reported school or sport-related events were not linked to specific states of aversive tension. After food intake, subsequent increases of aversive tension were diminished, and decreases of aversive tension were induced in adolescents with anorexia nervosa.
Kumar et al, 2018 [14]	N/A	GF^q^social, GFrole; BPRS^r^; Clinical Global Impression Severity; assorted questionnaires	At the end of project with repeat of clinical assessment	Daily survey completion rate was 41% and weekly survey completion rate was 39%	27 of 41 (66%) participants with early psychosis who completed the study and 11 of 13 (85%) treatment providers who responded to satisfaction surveys reported they would continue to use the app as part of treatment services.
Lim et al, 2020 [16]	N/A	SCID-5^s^, Positive and Negative Syndrome Scale, CDSS, Social Skills Performance Assessment, National Adult Reading Test, UCLA-LS^t^, SIAS^u^, Scales of Psychological Well-being	After treatment and 3-month follow up	Participants on average completed 95.47% of the +Connect (mean 40.10 days, SD 3.04)	Data indicate preliminary evidence that +Connect may reduce loneliness, with scores from preintervention (mean 52.58, SD 8.47) to postintervention (mean 48.10, SD 10.38) and at 3 months after the intervention (mean 42.89, SD 7.04) on UCLA-LS
Lim et al, 2019 [27]	11 lonely students without mental health conditions	SCID-5-Research version, UCLA-LS, SIAS, Centre for Epidemiological Studies-Depression	Posttreatment and 3-months follow up	Social anxiety group: 84.66%; control group: 90.26%	The UCLA-LS and straightforwardly worded SIAS scores decreased in a linear trend from baseline to months after the intervention for the case group. There were higher acceptability ratings across different ratings in a nonclinical lonely student group compared with those with social anxiety disorder.
Neumayr et al, 2019 [19]	50% were randomized to receive intervention	EDE-Q^v^, BMI, BDI-II^w^	8 weeks and 6 months	Mean of 231 logs during the 8-week intervention.	There were postintervention nonsignificant small to moderate between‐group effect sizes favoring the intervention group regarding BMI (*d*=–0.24 [–0.90, 0.41]) and eating disorder symptoms. At 6‐month follow‐up, there were no differences between the intervention group and control group
Niendam et al, 2018 [15]	N/A	BPRS	Not described	Weekly survey completion: mean 77.3%; Daily survey completion: mean 69.0%.	Weekly survey positive symptoms were significantly associated with BPRS-positive symptoms (β=.56, SE=0.10; *P*<.001).
Seidel et al, 2016 [18]	Healthy controls, normal weight, no history of eating disorders	Structured Interview for Anorexic and Bulimic Syndromes for Experts; Eating Disorder Inventory; BDI; Perseverative Thinking Questionnaire; State-Trait Anxiety Inventory; body weight	Not described	Participants answered 84.19% of their prompts compared to 75.73% in the control group	Momentary negative affect is positively associated with a higher amount of disorder-related rumination in participants (*P*<.001).
Shrier and Spalding, 2017 [20]	N/A	BDI-II	Not described	Not described	Participants expressed positive opinions about the ecological momentary intervention. They desired a high degree of personalization of the message quality, style, and voice.
Stallard et al, 2018 [26]	N/A	MFQ^x^; Revised Children’s Anxiety and Depression Scale; Strengths and Difficulties Questionnaire; Safety-assessment; Self-Harming information	After 2 weeks (postfamiliarization) and 12 weeks (after use)	Not described	73% of those who had recently self-harmed reported reductions in self-harm after using BlueIce for 12 weeks. There was a statistically significant mean difference of 4.91 (*P*=.04) on postuse symptoms of depression (MFQ) and 13.53 (*P*=.001) on symptoms of anxiety (Revised Child Anxiety and Depression Scale), which was evident across all anxiety subscales.
Sweidan et al, 2019 [23]	N/A	Not described	Not described	Not described	A detailed survey filled out by 100 parents and teachers after testing showed encouraging results

^a^TAU: treatment as usual.

^b^PANAS: Positive Affective and Negative Affective Scale.

^c^PSYRATS: Psychotic Symptom Rating Scale.

^d^CDSS: Calgary Depression Scale for Schizophrenia.

^e^EQ-5D-5L: 5-level health status and health-related quality of life.

^f^N/A: not applicable.

^g^MDD: major depressive disorder.

^h^PHQ-9: patient health questionnaire-9.

^i^HAM-D: Hamilton Depression Rating scale.

^j^HAM-A: Hamilton Anxiety Rating scale.

^k^GAIN-Q3: Global Appraisal of Individual Needs-Quick 3.

^l^EMA: ecological momentary assessment.

^m^SIQ-JHSV: Suicidal Ideation Questionnaire-Junior High School Version.

^n^CSSRS: Columbia Suicide Severity Rating Scale.

^o^CRAFT: Car, Relax, Alone, Forget, Friends, and Trouble questionnaire.

^p^ChEDE: child eating disorder examination.

^q^GF: global functioning.

^r^BPRS: Brief Psychiatric Rating Scale.

^s^SCID-5: Structured Clinical Interview for Diagnostic and Statistical Manual of Mental Disorders.

^t^UCLA-LS: University of California, Los Angeles Loneliness Scale score.

^u^SIAS: Social Interaction Anxiety Scale.

^v^EDE-Q: Eating Disorder Examination-Questionnaire.

^w^BDI-II: Beck Depression Inventory-II.

^x^MFQ: mood and feeling questionnaire.

### Treatment Delivered by the Apps

Eight of the studies included delivered treatment content in addition to and often in response to self-monitoring [13,16,19,20,24-27]. One of the studies used an app delivering educational content designed for children with autism and did not include any monitoring of symptoms or level of function [23]. The specific findings are presented in Table 4.

**Table 4:** Description of the treatments delivered by the apps in studies on self-monitoring and automatically generated data collected via smartphones in children, adolescents, and young adults with psychiatric disorders (n=9).

Author, year of publication	Intervention
Bucci et al, 2018 [13]	Actissist: Messages and cognitive or behavioral strategies aimed at ways of coping with distress; use of video, fact sheets, and external links. ClinTouch: only symptom monitoring.
Dennis et al, 2015 [24]	Participants had access to ecological momentary intervention content.
Kennard et al, 2018 [25]	Psychoeducation, behavioral activation and pleasant event scheduling, affect regulation strategies: savoring, switching, and distress tolerance, consolidation and review, distress tolerance strategies, emotion regulation skills, and safety plan.
Lim et al, 2020 [16] and Lim et al, 2019 [27]^a^	The app delivers positive psychology content daily.
Neumayr et al, 2019 [19]	Positive reinforcement, coping skills suggestions, motivational slogans, positive affirmations, guided meditations, and therapist feedback.
Shrier and Spalding, 2017 [20]	Messages of general support and messages to avoid sexual risk.
Stallard et al, 2018 [26]	Personalized mood-lifting activities and safety check to prevent self-harm.
Sweidan et al, 2019 [23]	The app delivers educational content in the following categories: numbers, vocabularies, letters, social skills, relaxation, and anger management

^a^This app was used in 2 different studies.

### Findings of the Studies

The majority of the included studies were feasibility or pilot studies. A total of 9 studies reported on acceptability and satisfaction specifically [13-16,19,24-27], with all studies reporting 70% or more of the participants stating they would recommend others to use the app or rating the app as helpful/useful or better. None of the included studies reported findings that would suggest the technology is not feasible. Specific findings are presented in Table 3.

### Findings of the Retrospective Cohort Study

One study described an app for self-monitoring of rumination about food and weight, as well as the self-assessment of valence, calmness, and energetic arousal [17]. The correlation between affect and negative rumination was investigated in a retrospective cohort study that compared the registrations from participants with anorexia nervosa and registrations from healthy controls. Analyses showed that for participants with anorexia nervosa, negative affect registered on the app was positively correlated with the amount of disorder-related rumination.

### Methodology and Findings of the RCTs

One RCT investigated the effectiveness of a self-monitoring system on participants with psychosis, thereby focusing on cognitive appraisals, belief convictions, emotions, and associated behaviors on a smartphone app [13]. The app used videos and fact sheets in combination with messages and cognitive or behavioral strategies aimed at coping with distress. The effect of this system was tested over a 12-week period on participants with early psychosis in an RCT that included 46 participants randomized in 2:1 to use the Actissist app for both self-monitoring and intervention, while the control group used an app with only self-monitoring. The primary outcome was feasibility and acceptability, and participants with early psychosis found the Actissist app to be both feasible and acceptable. The RCT also found a large treatment effect in relation to the secondary outcome, specified as an improvement of negative symptoms, general psychotic symptoms, and mood, as assessed by the Positive and Negative Syndrome Scale and Calgary Depression Scale for Schizophrenia.

The second RCT investigated the effectiveness of a self-monitoring system focusing on emotional distress, and the app responded with distress tolerance strategies, emotion regulation skills, and a safety plan [25]. The effect of this system was tested in an RCT on participants hospitalized for suicidal ideation or a recent suicide attempt. This included 66 participants randomly organized into treatment and control groups in a 1:1 ratio, with the treatment group receiving the intervention app, while the control group received treatment as usual over the course of 2-3 weeks. Analyses based on their primary outcomes showed that the treatment had no effect on suicidal ideation.

The third RCT investigated the effectiveness of a self-monitoring system focusing on meals, feelings, thoughts, and behavior, where the app responded with positive reinforcement, coping skills suggestions, motivational slogans, positive affirmations, guided meditations, and therapist feedback [19]. The effect of this system was tested in an RCT on participants with anorexia nervosa, including 40 participants randomly organized into treatment and control groups in a 1:1 ratio, with the treatment group receiving the intervention app while the control group received treatment as usual over the course of 8 weeks. Analyses on the primary outcome concerning feasibility suggested that the intervention was both feasible and acceptable, at least in the short term in combination with feedback from therapists. Analyses based on secondary outcomes showed nonsignificant differences favoring the intervention group in the normalizing of the participant’s body mass index.

### Risk of Bias and Quality of Evidence Assessment

**Figure 2:**
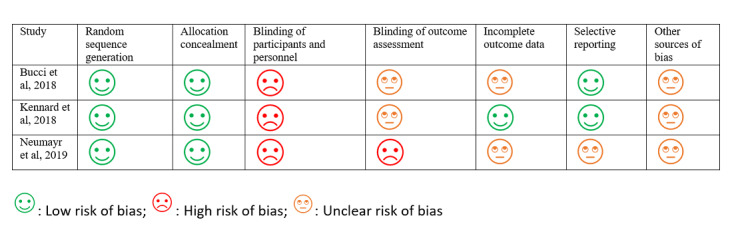
Cochrane risk of bias assessment chart for the included randomized controlled trial studies.

**Figure 3:**
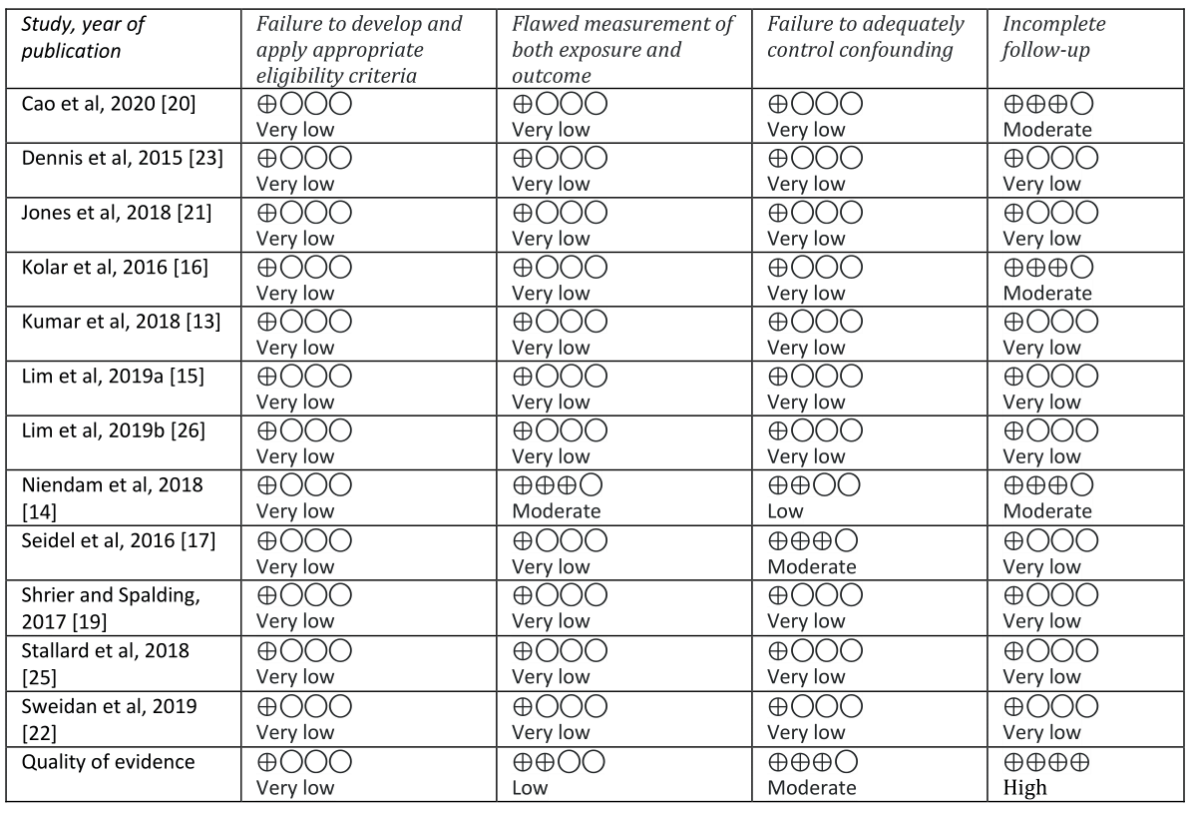
Grading of Recommendations, Assessment, Development and Evaluations (GRADE) quality of evidence chart for nonrandomized studies.

Of the 3 RCTs, only 1 conducted an intention-to-treat analysis [25]; the remaining 2 [13,19] had dropouts but did not describe how these were handled in the analyses. Two studies described being conducted in accordance with a predefined protocol; the protocol is included in the reference list [13,25], and the remaining 1 did not mention following a specific protocol. In 2 studies, the researchers were blinded for the randomization [13,25] but the participants were not blinded for any of the studies. All the studies described randomization processes with a low risk of bias; however, all studies were evaluated to have an unclear risk of bias due to other sources. One of the studies mentioned their design’s lack of ability to determine which components of the intervention or app were effective as a limitation [25]. All the nonrandomized studies were evaluated to have low quality of evidence, mainly due to the lack of controls. Results from the Cochrane Risk of Bias assessment and the GRADE assessment of quality of evidence are presented in Figure 2 and Figure 3, respectively.

### Declarations

In 9 of the included studies, conflicts of interest were disclosed [14,16,18-21,23,26,27]. Six of these studies disclosed no relevant conflicts of interest, and in 3 studies [19,21,26], 1 of the authors of the study was also the designer of the app used in the study.

## Discussion

### Principal Findings

Despite the fact that 95% of the teens in the United States own and use a smartphone, we were able to identify only 15 unique studies using smartphone-based self-monitoring and treatment for 8 different diagnostic groups in children, adolescents, and young adults with psychiatric disorders. The included studies were highly heterogeneous in terms of the aims of the study, the included participants, the methodology used, and the reporting of the findings. The fact that 11 out of the 15 included studies were published during the last 3 years demonstrates that the use of smartphone-based health technology for children, adolescents, and young adults with mental health problems is still in an early stage. Although all the studies used smartphones for self-monitoring or treatment, only 3 RCTs with relatively small sample sizes that investigated the effectiveness of smartphone-based intervention treatments have been published. Of these, 2 found a positive treatment effect and the third showed no effect. However, 2 of these studies had feasibility and acceptability as the primary outcome measure, and all the RCTs had several issues concerning a high or unclear risk of bias. In general, the effectiveness of smartphone-based treatment for children, adolescents, and young adults with various psychiatric disorders has been sparingly investigated and is yet undetermined. Despite the great potential of smartphone-based monitoring and treatment, more RCTs investigating the potential positive and negative effects of using smartphones to deliver health interventions in this population are required.

The majority of the studies identified in this systematic review were feasibility or pilot studies, with the main findings describing different aspects of the acceptance, usability, and feasibility of smartphone-based self-monitoring during generally quite short study periods or different lengths. The vast majority of these studies reported positive attitudes among participants regarding the acceptance and feasibility of self-monitoring information. Notably, only 1 of the included studies reported on the validity of the various self-monitored data as compared with the validated rating scales such as the Hamilton Depression Rating Scale [28]. Therefore, the validity of the self-monitored data collected in the included studies is yet undetermined. In 2 studies, automatically generated data were collected. Both collected data on location and usage. One of them collected data on step count and ambient light and investigated the correlation between automatically generated data and clinical rating scores. It may be that some of the studies collected automatically generated data but did not include or mention it in the respective studies.

### Limitations of the Individual Studies

Only 1 RCT described strictly monitoring, reviewing, and documenting any serious adverse effects of the intervention [13]. None of the other RCTs conducted a systematic assessment of the potential adverse effects of the intervention. The generalizability of the results is questionable as none of the included studies investigated the large-scale use of smartphone-based treatment in daily clinical practices, and all but 1 of the studies were conducted in developed countries. Only 9 of the 15 studies disclosed a potential conflict of interest. In 6 of these studies, there were no relevant conflicts of interest, and in the remaining 3, one of the authors of the study designed the app used in the study. However, they did not receive any financial gain from its development. The findings of the studies were reported in a number of different ways, especially with regard to reporting the participant adherence to self-monitoring. In all of the studies that reported the adherence to self-reporting, each study had its own definition of completion of a task and how to report this as adherence; it would be greatly beneficial if this could be done in a more homogenous and standardized way in order to facilitate comparisons and meta-analyses. The participants’ clinical diagnoses were validated by the researchers in only 2 of these studies. Only 1 of the 3 RCTs used an intention-to-treat analysis, and in the remaining 2, it was not reported how dropouts were handled. Only 2 were single-blinded, and none were double-blinded.

### Limitations and Strengths of This Review

The studies included in this systematic review were heterogeneous both in the clinical profiles of the participants and in the methodologies, making it difficult to compare the results and draw legitimate conclusions. Because we were interested in describing studies performed on clinical populations, we chose to only perform the literature search in databases dedicated to medical and psychological publications. Therefore, we may have missed some eligible studies that were only published in technology-oriented journals or conference proceedings or literature that may have been identified by a grey literature search. Because the included studies presented with a number of different ways of securing or assessing the diagnosis in their clinical populations, we were not able to create strict inclusion criteria regarding diagnostic assessments according to standardized diagnostic interviews. However, we chose to include only studies where participants were referred by an external clinician who had established the diagnosis or need for treatment. Thus, we only included populations with a psychiatric diagnosis. Further, it is important to mention that the inclusion criteria for the diagnostic foundation of the participants were clarified during the review process to also include studies with participants with severe symptoms requiring treatment, for example, suicidal behavior and self-harm behavior. This specification regarding the clinical status of the participants was made to ensure identification of all relevant studies for the review. Similarly, the exclusion criteria were slightly specified during the review process adding that studies including “people with symptoms not meeting diagnostic criteria” to criterion 1. We do find that the inclusion and exclusion criteria were predefined as good as possible and further clarified during the review process, and the review therefore has been conducted in accordance with the principles of the Cochrane Handbook for Systematic Reviews of Interventions. Further, it would be interesting for future reviews to include studies investigating the use of smartphone-based self-assessment, treatment, and automatically generated data in populations at risk of developing psychiatric disorders or in populations with subclinical symptoms. During the review process, we decided to include studies investigating smartphone-based treatment, in addition to only studies involving monitoring, which was the original criterium; this was done because we saw many of the monitoring apps also provided treatment, and thus, it was meaningful to describe both monitoring and treatment in the same review. This review was performed with a systematic approach and we conducted a thorough investigation of more than 2000 potential studies. Even though this review has 4 authors, only SM performed the eligibility screening and the risk of bias assessment. The literature search was updated throughout the process, and the results reflect an updated review of the existing literature. This review was not preregistered in any prospective review databases (eg, PROSPERO). The authors of this systematic review are experts within the field on both the research and clinical sides. All of the authors are involved in a Horizon 2020 project named Technology Enabled Mental Health-Innovation Training Network, which focuses on technology-based solutions to improve the assessment, prevention, and treatment of mental health disorders in children, adolescents, and young adults [30]. The studies included in this review covered a broad range of diagnostic groups and thus provided a good overview of the current research published within this rapidly expanding field.

### Conclusions and Implications

This systematic review identified 15 individual studies examining the use of smartphone-based monitoring and treatment of children, adolescents, and young adults with psychiatric disorders, who were referred by external clinicians, thereby covering 8 different diagnostic categories. This review identified a large diversity in the research conducted in the field of smartphone-based self-monitoring and treatment of children, adolescents, and young adults with psychiatric disorders. Most of the included studies were feasibility or pilot studies, and only 3 RCTs investigating the effect of smartphone-based treatment were identified. This review demonstrates that for children, adolescents, and young adults with psychiatric disorders, adherence to smartphone-based symptom registration was generally high, as was the satisfaction with such a system, as reported by participants, clinicians, and caregivers. Among the 3 RCTs, 2 found a positive treatment effect, which is promising for the future of technological interventions in the mental health of children, adolescents, and young adults with psychiatric disorders. However, all the 15 studies were short-term studies and generally had small sample sizes, with an average of 43 participants.

In conclusion, the findings from this review strongly emphasize the need for a larger number of studies as well as studies with a larger number of participants, including RCTs investigating the potential positive and negative effects of fine-grained smartphone-based self-monitoring and treatment over prolonged periods of time. Such RCTs should provide details on the methodology, reporting, and interpretation of findings, as recently described by our group [31], thereby making it easier to compare studies and to facilitate future meta-analyses.

Smartphones represent a fine-grained, unobtrusive, and effective way to monitor symptoms and level of function that could help distinguish severe psychiatric health problems from normal behavior. This could potentially lead to more efficient use of clinical resources within today’s health care system, which in turn can lead to the more equitable distribution of resources. One of the studies in this review used a model where the caregiver of the child reported the symptoms, which might help parents/caregivers observe their child in a more systematic manner. Children, adolescents, and young adults often have well-established behaviors regarding smartphone usage, which suggests that in order to help them engage with mental health apps, the apps need to be designed to fit their habits and be customizable to their needs [32]. In this way, smartphones hold great potential as a modern and widely available technology platform for psychiatric care, especially as children, adolescents, and young adults can be reluctant to seek professional help due to the stigma and negative attitudes toward mental health problems [33].

## Appendix

Multimedia Appendix 1 PRISMA 2009 checklist.

## Abbreviations

GRADE: Grading of Recommendations, Assessment, Development and Evaluations
PRISMA: Preferred Reporting Items for Systematic Reviews and Meta-Analysis
RCT: randomized controlled trial

## References

[1] Kessler RC, Berglund P, Demler O, Jin R, Merikangas KR, Walters EE (2005). Lifetime prevalence and age-of-onset distributions of DSM-IV disorders in the National Comorbidity Survey Replication. Arch Gen Psychiatry.

[2] Adolescent Mental Health. World Health Organization.

[3] McGorry P (2011). Transition to adulthood: the critical period for pre-emptive, disease-modifying care for schizophrenia and related disorders. Schizophr Bull.

[4] Shiffman S, Stone AA, Hufford MR (2008). Ecological momentary assessment. Annu Rev Clin Psychol.

[5] Number of smartphone users worldwide from 2016 to 2021. Statista (2020).

[6] Anderson M, Jiang J Pew Research Center (2018) Internet and Technology.

[7] Huguet A, Rao S, McGrath PJ, Wozney L, Wheaton M, Conrod J, Rozario S (2016). A Systematic Review of Cognitive Behavioral Therapy and Behavioral Activation Apps for Depression. PLoS One.

[8] Wang K, Varma DS, Prosperi M (2018). A systematic review of the effectiveness of mobile apps for monitoring and management of mental health symptoms or disorders. J Psychiatr Res.

[9] Moher D, Liberati A, Tetzlaff J, Altman DG, PRISMA Group (2009). Preferred reporting items for systematic reviews and meta-analyses: the PRISMA statement. BMJ.

[10] Recognizing Adolescence. World Health Organization.

[11] Julian TPH, Sally G (2009). Cochrane Handbook for Systematic Reviews of Interventions Version 5.1.0. The Cochrane Collaboration, 2011.

[12] Guyatt G, Oxman A, Schünemann Holger J, Tugwell P, Knottnerus Andre (2011). GRADE guidelines: a new series of articles in the Journal of Clinical Epidemiology. J Clin Epidemiol.

[13] Bucci S, Barrowclough C, Ainsworth J, Machin M, Morris R, Berry K, Emsley R, Lewis S, Edge D, Buchan I, Haddock G (2018). Actissist: Proof-of-Concept Trial of a Theory-Driven Digital Intervention for Psychosis. Schizophr Bull.

[14] Kumar D, Tully LM, Iosif A, Zakskorn LN, Nye KE, Zia A, Niendam TA (2018). A Mobile Health Platform for Clinical Monitoring in Early Psychosis: Implementation in Community-Based Outpatient Early Psychosis Care. JMIR Ment Health.

[15] Niendam TA, Tully LM, Iosif A, Kumar D, Nye KE, Denton JC, Zakskorn LN, Fedechko TL, Pierce KM (2018). Enhancing early psychosis treatment using smartphone technology: A longitudinal feasibility and validity study. J Psychiatr Res.

[16] Lim MH, Gleeson JFM, Rodebaugh TL, Eres R, Long KM, Casey K, Abbott JM, Thomas N, Penn DL (2020). A pilot digital intervention targeting loneliness in young people with psychosis. Soc Psychiatry Psychiatr Epidemiol.

[17] Kolar D, Hammerle F, Jenetzky E, Huss M, Bürger Arne (2016). Aversive tension in female adolescents with Anorexia Nervosa: a controlled ecological momentary assessment using smartphones. BMC Psychiatry.

[18] Seidel M, Petermann J, Diestel S, Ritschel F, Boehm I, King JA, Geisler D, Bernardoni F, Roessner V, Goschke T, Ehrlich S (2016). A naturalistic examination of negative affect and disorder-related rumination in anorexia nervosa. Eur Child Adolesc Psychiatry.

[19] Neumayr C, Voderholzer U, Tregarthen J, Schlegl Sandra (2019). Improving aftercare with technology for anorexia nervosa after intensive inpatient treatment: A pilot randomized controlled trial with a therapist-guided smartphone app. Int J Eat Disord.

[20] Shrier LA, Spalding A (2017). "Just Take a Moment and Breathe and Think": Young Women with Depression Talk about the Development of an Ecological Momentary Intervention to Reduce Their Sexual Risk. J Pediatr Adolesc Gynecol.

[21] Cao J, Truong AL, Banu S, Shah AA, Sabharwal A, Moukaddam N (2020). Tracking and Predicting Depressive Symptoms of Adolescents Using Smartphone-Based Self-Reports, Parental Evaluations, and Passive Phone Sensor Data: Development and Usability Study. JMIR Ment Health.

[22] Jones RM, Tarpey T, Hamo A, Carberry C, Lord C (2018). Smartphone measures of day-to-day behavior changes in children with autism. NPJ Digit Med.

[23] Sweidan S, Salameh H, Zakarneh R, Darabkh KA (2019). Autistic Innovative Assistant (AIA): an Android application for Arabic autism children. Interactive Learning Environments.

[24] Dennis ML, Scott CK, Funk RR, Nicholson L (2015). A Pilot Study to Examine the Feasibility and Potential Effectiveness of Using Smartphones to Provide Recovery Support for Adolescents. Subst Abus.

[25] Kennard B, Goldstein T, Foxwell A, McMakin D, Wolfe K, Biernesser C, Moorehead A, Douaihy A, Zullo L, Wentroble E, Owen V, Zelazny J, Iyengar S, Porta G, Brent David (2018). As Safe as Possible (ASAP): A Brief App-Supported Inpatient Intervention to Prevent Postdischarge Suicidal Behavior in Hospitalized, Suicidal Adolescents. Am J Psychiatry.

[26] Stallard P, Porter J, Grist R (2018). A Smartphone App (BlueIce) for Young People Who Self-Harm: Open Phase 1 Pre-Post Trial. JMIR Mhealth Uhealth.

[27] Lim MH, Rodebaugh TL, Eres R, Long KM, Penn DL, Gleeson JFM (2019). A Pilot Digital Intervention Targeting Loneliness in Youth Mental Health. Front Psychiatry.

[28] Shahid A, Wilkinson K, Marcu S, Shapiro CM (2011). Hamilton Rating Scale for Depression (HAM-D). STOP, THAT and One Hundred Other Sleep Scales.

[29] Hamilton M (1959). The assessment of anxiety states by rating. Br J Med Psychol.

[30] TEAM ITN (2019). Technology Enabled Mental Health.

[31] Tønning Morten Lindbjerg, Kessing LV, Bardram JE, Faurholt-Jepsen M (2019). Methodological Challenges in Randomized Controlled Trials on Smartphone-Based Treatment in Psychiatry: Systematic Review. J Med Internet Res.

[32] Michel T (2019). An explorative review of youth mental health apps for prevention and promotion. PervasiveHealth - EAI.

[33] Rickwood D, Deane F, Wilson CJ (2007). When and how do young people seek professional help for mental health problems?. Medical Journal of Australia.

